# Positive Antinuclear Antibody (ANA)-Negative Systemic Lupus Erythematosus (SLE) Presenting With Acute Pancreatitis

**DOI:** 10.7759/cureus.23031

**Published:** 2022-03-10

**Authors:** Joydeep Samanta, Souveek Mitra, Kaushik Sen, Avishek Saha

**Affiliations:** 1 Rheumatology, Institute of Medical Science and SUM Hospital, Bhubaneswar, IND; 2 Hepatology, Institute of Post Graduate Medical Education & Research, Kolkata, IND; 3 Internal Medicine, Nilratan Sircar Medical College, Kolkata, IND; 4 Cardiology, Nilratan Sircar Medical College, Kolkata, IND

**Keywords:** systemic lupus erythematosus, acute cutaneous lupus erythematosus, subacute cutaneous lupus erythematosus, acute pancreatitis, ana negative

## Abstract

Systemic lupus erythematosus (SLE) is an autoimmune disease with multisystem involvement and most commonly affects women of childbearing age. Most of the patients, if not all, have positive antinuclear antibody (ANA) in their serum. ANA-negative SLE is extremely rare. Here, we present a case of a 15-year-old girl presenting with pancreatitis due to ANA-negative SLE.

## Introduction

Systemic lupus erythematosus (SLE) is an autoimmune disease with multisystem involvement that most commonly affects women of childbearing age. Most of the patients, if not all, have positive antinuclear antibody (ANA) in their serum. As per the 2019 European League Against Rheumatism/American College of Rheumatology (EULAR/ACR) classification criteria, ANA positivity is an entry criterion for the diagnosis of SLE [1]. ANA-negative SLE is extremely rare, more so over with recent advancements of diagnostic methods and laboratory techniques. We hereby present a case of a 15-year-old girl presenting with pancreatitis due to ANA-negative SLE, which, in our belief, is really rare in modern days.

## Case presentation

A 15-year-old girl presented with pain abdomen for three days, along with mildly itchy, erythematous skin rash over the face and discoid-shaped rashes all over the trunk for one month. Her abdominal pain was moderate in intensity, being more severe around the periumbilical region and radiating to the back. The pain didn’t have any definite relationship with food intake, however, due to severe pain along with a few episodes of non-bilious vomiting, she was not taking much orally for the last two days. There was no history of hematemesis, melena, or diarrhea. Her facial rashes were mildly itchy and photosensitive, along with rashes over the upper trunk. She also complained of recurrent painless oral ulcers for the last six months. On further inquiry, she gave the history of a sense of weakness for the last two weeks with difficulty while getting up from the sitting position but without any difficulty in doing an overhead activity. She didn’t have any history of fever, joint symptoms, or urinary symptoms. There was no history of any drug intake in the recent past.

**Figure 1:**
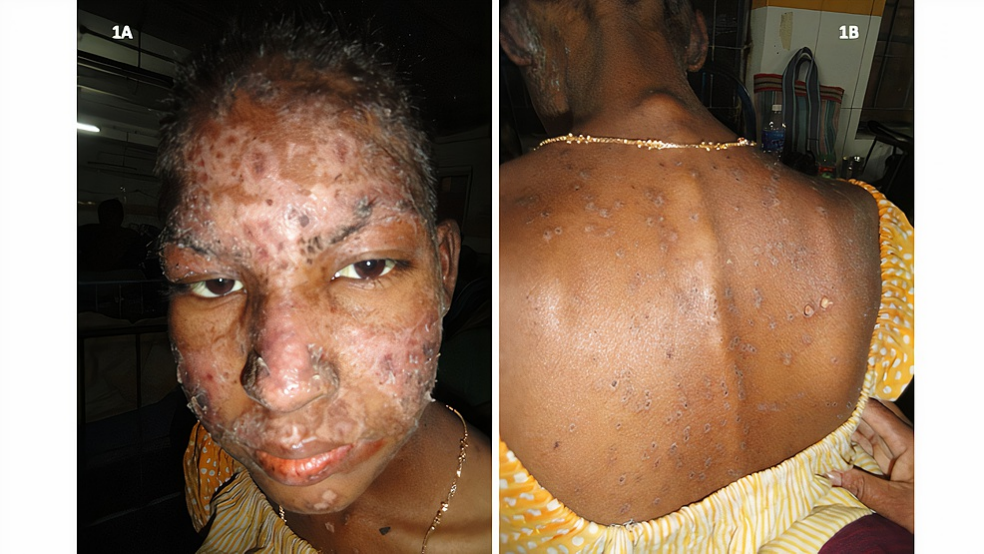
Cutaneous rash of the patients showing features of cutaneous lupus erythematosus 1A: Erythematous rash with scaling over the face, especially over the malar region with distinct sparing of nasolabial folds 1B: Discoid-shaped rashes over the trunk with heaped-up scales noted over a few rashes

Physical examination revealed mild pallor, mild bipedal edema up to the ankles, few small ulcers over the soft palate, erythematous rash over the malar region with distinct sparing of nasolabial folds, and discoid-shaped rashes over the trunk with heaped-up scales noted over a few rashes (Figures 1A-1B).

The abdominal examination revealed generalized tenderness over the abdomen, more in the umbilical region without any organomegaly. Besides, neurological examination revealed Medical Research Council (MRC) grade 4/5 power in the proximal group of muscles in both the upper and lower limbs with 5/5 power in the distal group of muscles, no truncal or neck weakness, normal deep tendon reflexes, and normal sensory examination. The rest of the systemic examinations were non-revealing.

**Figure 2:**
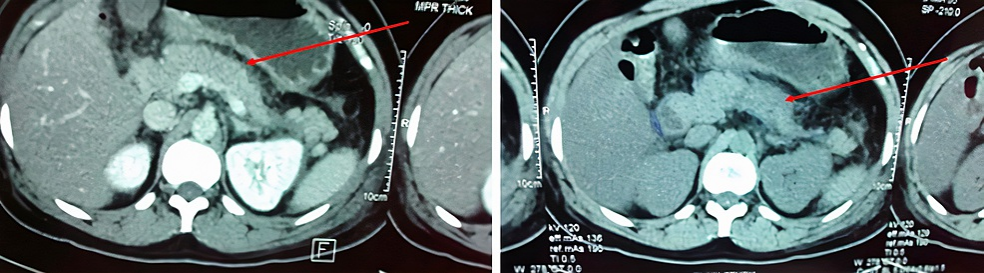
Contrast-enhanced CT (CECT) abdomen of patients 2A and 2B: CECT abdomen showing bulky pancreas with peripancreatic inflammatory stranding and fluid collection suggestive of acute pancreatitis

Her blood investigations revealed bicytopenia (anemia and thrombocytopenia), high erythrocyte sedimentation rate (ESR), normal C-reactive protein, transaminitis (serum glutamic-oxaloacetic transaminase(SGOT)>serum glutamate-pyruvate transaminase(SGPT)), elevated muscle enzymes (creatine phosphokinase, lactate dehydrogenase), and elevated serum amylase and lipase. The renal function test was normal, however, urine microscopy showed the presence of white and red blood cells with trace proteinuria. Both blood and urine cultures were sterile, and serum procalcitonin came out to be normal. Contrast-enhanced computed tomography (CECT) of the chest and abdomen showed bilateral mild pleural effusion, bulky pancreas, with peripancreatic inflammatory stranding and fluid collection suggestive of acute pancreatitis (Figures 2A-2B).

Echocardiography showed mild pericardial effusion with good left ventricular function in the absence of any valvular abnormality or vegetations. In suspicion of autoimmune etiology, further workup was done. Anti-nuclear antibody (ANA) by indirect immunofluorescence came out to be negative even on serial dilution, however, the anti-Ro antibody was strongly positive on line blot assay, with anti-dsDNA, anti-Sm, and anti-La all being negative. Serum complements were low. All myositis-specific antibodies also came out to be negative. Routine blood investigation parameters are being shown in Table 1.

**Table 1:** Blood investigation reports SGPT - Serum glutamic-pyruvic transaminase; SGOT - Serum glutamic-oxaloacetic transaminase; ALP - Alkaline phosphatase; CPK - Creatine phosphokinase; LDH - Lactate dehydrogenase

Parameters	Value
Haemoglobin (gm/dL) (12.0-16.0 gm/dL)	7.2
Total leukocyte count (10^9^/L) (4.0-11.0 x 10^9^/L)	6.5
Differential count	N-69% L-23% M-01% E-%09 B-00%
Platelet count (10^9^/L) (150-450 x 10^9^/L)	80
Erythrocyte sedimentation rate (2-20 mm/1^st^ hour)	140 mm/1^st ^hr
Packed cell volume/hematocrit (%) (36-46%)	21.6
Mean corpuscular volume (fl) (80-100 fl)	93.6
Mean corpuscular hemoglobin (pg) (26-34 pg)	30.9
Mean corpuscular hemoglobin concentration (gm/dL) (31-37 gm/dL)	33.5
Serum bilirubin (mg/dL) (0.2-1.2 mg/dL)	1.0
SGPT (U/L) (2-41 U/L)	68
SGOT (U/L) (2-40 U/L)	312
ALP (U/L) (40-128 U/L)	126
Total protein (gm/dL) (6.0-8.3 g/dL)	5.3
Albumin (gm/dL) (3.4-4.8 g/dL)	3.0
CPK (U/L) (26-192 U/L)	455
LDH (U/L) (0-248 U/L)	779
Serum urea (mg/dL) (10-50 mg/dL)	20.9
Serum creatinine (mg/dL) (0.5-1.2 mg/dL)	0.9

With these available investigations, autoimmune aetiologies like systemic lupus erythematosus (SLE) or hypcomplementemic urticarial vasculitis syndrome (HUVS) were considered the possible differentials. Although multisystem involvement in the form of cutaneous, hematological (bicytopenia), musculoskeletal (myositis), gastrointestinal (pancreatitis), and renal (proteinuria) was favoring a diagnosis like SLE, ANA negativity, even on higher dilution, was going against a diagnosis of SLE. On the other hand, HUVS could be a close mimicker of SLE. Serum C1q level was done, which came out to be normal. A skin biopsy was done, which was suggestive of subacute cutaneous lupus erythematosus. Also, based on the presence of trace proteinuria at baseline in the urine routine microscopy, further quantification was done, which revealed 24 hours proteinuria of 0.78 gm. Renal biopsy was done, which revealed mesangial hypercellularity with the mesangial immune deposit, suggestive of class II lupus nephritis. So, although ANA was negative even at higher dilution, based on skin biopsy and renal biopsy findings, a diagnosis of SLE was made.

Initially, a broad-spectrum antibiotic (ceftriaxone) was started considering the possibility of urinary tract infection due to the presence of pus cells in urine routine microscopy. With high clinical suspicion of autoimmune etiology, she was also started on oral corticosteroid (prednisolone 1 mg/kg/day). Later, after the urinary culture came out to be sterile, intravenous methylprednisolone pulse (500 mg daily for three days) was given followed by the continuation of oral steroid. The patient responded to steroids dramatically. Urine became clear within three days, and pain in the abdomen subsided. She was also started on monthly intravenous cyclophosphamide pulse as per the modified National Institutes of Health (NIH) regimen (0.50 mg/m2).

## Discussion

SLE is a systemic autoimmune disease with ANA positivity being considered Sina quo non nowadays. There were some anecdotal reports of ANA-negative SLE, but those were mostly due to technical inaccuracy like the use of mouse or rat tissue as substrate and the use of ethanol or methanol as a fixating agent. However, after the invention of the HEp-2 cell line, ANA-negative SLE has become exceedingly rare. McHardy et al. reported that 9% of SLE patients tested negative for ANA when rat liver was used as the substrate [2-3]. But, Worrall et al. found that only 2% of SLE patients tested negative for ANA when the test was conducted using HEp-2 cells [4]. Apart from the technical inaccuracy previously described, the prozone phenomenon can be another important cause of ANA negativity even on the HEp-2 cell line, which can be circumvented by testing ANA in serial dilution [5].

Overall. ANA-negative SLE is exceedingly rare, which represents 1-5% of total cases of SLE. Amongst those cases, the anti-Ro antibody is commonly positive [6-7]. In our case, ANA was negative in the HEp-2 cell line in serial dilution, thus excluding the possibility of any technical inaccuracy/prozone phenomenon. However, anti-Ro positivity and histopathological evidence helped clinch the diagnosis in our case.

In our case, the girl presented with skin rash, bicytopenia, polyserositis (pleural, pericardial effusion, and ascites), high ESR, and low C3 and C4. All these manifestations can be seen both in SLE and hypocomplementemic urticarial vasculitis syndrome (HUVS) [8]. Moreover, renal involvement has also been reported in HUVS. Both these conditions have low serum complements. However, ANA positivity usually differentiates between these two conditions. Although ANA was negative in our case, there were other manifestations like pancreatitis, myositis which are not seen in HUVS. Besides, the C1q level is usually low in HUVS, which was normal in our case, thus making HUVS a less likely possibility.

Among the various organ manifestations of SLE, musculoskeletal and mucocutaneous involvement is the commonest followed by renal involvement. Myositis is a relatively less common musculoskeletal manifestation, being seen in around 10% of cases. Among the various types of gastrointestinal involvement of SLE, acute pancreatitis is relatively rare. Reynolds et al. reviewed hospitalized SLE patients and found acute pancreatitis in 20 of 241 patients (8.2%) [9]. Gallstones and alcohol are responsible for about 80% of cases of acute pancreatitis. Glucocorticoids used for treatment can precipitate pancreatitis sometimes. SLE disease activity per se may be responsible in some cases. The pathogenic mechanism of SLE-related acute pancreatitis is very complex and multifactorial. Vascular damage (including vasculitis, intimal thickening, immune complex deposition, occlusion of arteries, and arterioles), autoantibody production, abnormal cellular immune response, and drug toxicity may be responsible for the development of pancreatitis [10]. Treatment is directed against the control of underlying SLE disease activity. In our case also, the patient presented with myositis and pancreatitis along with cutaneous and renal involvement. Such a florid presentation of SLE with ANA being negative is relatively rare. Unless it is diagnosed on time, it can be life-threatening.

## Conclusions

To conclude, although ANA positivity is an entry criterion as per the latest EULAR/ACR classification criteria for SLE, rarely, ANA-negative SLE may present. In such a scenario, clinical presentation along with histopathological evidence may help clinch the diagnosis.
